# Supplementary material for: Complexome profiling of the Chlamydomonas psb28 mutant reveals TEF5 as an early PSII assembly factor
Source: Plant Cell. 2025 Mar 17;37(6):koaf055. doi: 10.1093/plcell/koaf055 (PMC12164590; doi:10.1093/plcell/koaf055)
Supplement: koaf055_Supplementary_Data [file koaf055_supplementary_data.zip › psb28_interactive.zip!/plots/intensity/qualityPlots/peptides/ATP6_Cre01.g018800.t1.2.html]

### Cre01.g018800.t1.2

Trivial name: ATP6  
SeqCoverage [median]: 10.00  
Mass: 35.51 kDa  
  
 

| Sequence | MissedCleavage | Mass [Da] | wtIntensity | muIntensity | Score |
| --- | --- | --- | --- | --- | --- |
| AVSLGVR | 0 | 700.42 | 30187.71 | 71505.01 | 148.28 |
| MLPAIVDASDNR | 0 | 1300.64 | 75489.43 | 126141.23 | 204.64 |
| SQAPMDKPHAPVDDR | 1 | 1662.78 | 57940.10 | 82950.87 | 136.26 |
|  |  |  | 163617.2 | 280597.1 |  |
